# An Empirical Comparative Study on the Two Methods of Eliciting Singers’ Emotions in Singing: Self-Imagination and VR Training

**DOI:** 10.3389/fnins.2021.693468

**Published:** 2021-08-12

**Authors:** Jin Zhang, Ziming Xu, Yueying Zhou, Pengpai Wang, Ping Fu, Xijia Xu, Daoqiang Zhang

**Affiliations:** ^1^College of Arts, Nanjing University of Aeronautics and Astronautics, Nanjing, China; ^2^MIIT Key Laboratory of Pattern Analysis and Machine Intelligence, College of Computer Science and Technology, Nanjing University of Aeronautics and Astronautics, Nanjing, China; ^3^Department of Library Services, Central Washington University, Ellensburg, WA, United States; ^4^Department of Psychiatry, Affiliated Nanjing Brain Hospital, Nanjing Medical University, Nanjing, China

**Keywords:** vocal music teaching, singing emotion, self-imagination, virtual reality, electroencephalogram, emotion classification

## Abstract

Emotional singing can affect vocal performance and the audience’s engagement. Chinese universities use traditional training techniques for teaching theoretical and applied knowledge. Self-imagination is the predominant training method for emotional singing. Recently, virtual reality (VR) technologies have been applied in several fields for training purposes. In this empirical comparative study, a VR training task was implemented to elicit emotions from singers and further assist them with improving their emotional singing performance. The VR training method was compared against the traditional self-imagination method. By conducting a two-stage experiment, the two methods were compared in terms of emotions’ elicitation and emotional singing performance. In the first stage, electroencephalographic (EEG) data were collected from the subjects. In the second stage, self-rating reports and third-party teachers’ evaluations were collected. The EEG data were analyzed by adopting the max-relevance and min-redundancy algorithm for feature selection and the support vector machine (SVM) for emotion recognition. Based on the results of EEG emotion classification and subjective scale, VR can better elicit the positive, neutral, and negative emotional states from the singers than not using this technology (i.e., self-imagination). Furthermore, due to the improvement of emotional activation, VR brings the improvement of singing performance. The VR hence appears to be an effective approach that may improve and complement the available vocal music teaching methods.

## Introduction

The generation of human emotion has a certain regularity that reflects the degree of experience and cognitive relationship between objectivity and subjectivity (Xi, 2010). Singing is a performing art that uses sound as a tool to awaken the human spirit, which fully embodies the self-creation and pursuit of human emotional expression (Xu, 2008). The art of singing is the art of human mood. The singing activities of singers are the expression of their inner psychology. These activities are inseparable from important psychological factors such as feeling, perception, consciousness, will, memory, imagination, emotion, and thinking (Mu, 2011). Singers need to pay attention to their emotional expression, truly integrate their thoughts and feelings with the song, and give life and soul to the song (Shi, 2002). Therefore, in the process of learning vocal music, students not only need to master the vocalization skills, but also need to actively invest in emotions, effectively express the inner emotions of musical works, and give them vitality and appeal.

Current vocal singing learning in Chinese universities is mainly based on the teaching of vocal theories and knowledge. The traditional self-imagination training for vocal learners does not efficiently engage and attracts students’ attention. We have observed that vocal music singers in our institution have insufficient perception and lack the ability to sing emotionally. In the process of song interpretation, the emotional expression of the singers is not ideal, and the emotional state is not actively mobilized. Thus it is difficult to achieve the requirements of the singing state with a strong voice.

Immersive virtual reality (VR) technology has existed for 50 years (Slater, 2009) and has been regarded as a promising and clinical tool (Kourtesis et al., 2019a). It enables researchers to collect advanced cognitive and behavioral data through dynamic stimuli and interactions in an ecologically valid environment, and it can be combined with non-invasive brain imaging techniques (Kourtesis et al., 2020). Virtual environments are sophisticated human-computer interfaces that can be used for a wide variety of applications (Skarbez et al., 2017). In recent years, VR technology has greatly met the needs of art education due to its multi-sensing, immersive, interactive, and imaginative characteristics. Though it has been widely used in design (Huang and Chen, 2017; Pei, 2017) and art education (Gao, 2013; Zhang, 2018), VR training has scarcely been used in the field of vocal teaching performance. Vocal music requires a comprehensive response of multiple psychological factors such as perception, thinking, imagination, and movement.

An emotion is a subjective state of being that we often describe as our feelings that impact human behavior and mental health (Zhang G. H. et al., 2019). The neuroscience research shows that the trigger of emotion is closely related to physiological activities, especially brain activities (LeDoux, 2000), which provides a theoretical basis for identifying emotional states by analyzing brain activities. Electroencephalogram (EEG) signal has the advantages of high time resolution, portability, and non-invasiveness (Baig and Kavakli, 2018). Emotion recognition based on EEG has received widespread attention (Alarcao and Fonseca, 2017). Generally, researchers collect EEG data during the elicitation of emotions by presenting videos, pictures, and other emotionally stimulating means. Then they extract relevant EEG features to explore the correlation between EEG features and different emotion categories (Hajcak et al., 2010). Some studies use machine learning algorithms to predict emotion based on EEG features (Wang et al., 2014; Li et al., 2019). Today EEG-based emotion recognition has been utilized in the rehabilitation treatment of patients with impaired consciousness (Huang et al., 2019), soldier mental state assessment (Lin et al., 2017), driving status monitoring (Halim and Rehan, 2020), but has not yet used in the area of eliciting singers’ emotion in singing.

In recent years, VR combined with EEG has been adopted in rehabilitation (Calabrò et al., 2017), stress relief (Tarrant et al., 2018), and teaching (Sood and Singh, 2018). Also, immersive VR and brainwave technologies have been adopted across education and training fields (Yang and Ren, 2019).

Inspired by these researches, an empirical study of combining VR training and EEG to investigate the effect of the method in eliciting emotions was designed. It is important to emphasize that combined VR training and EEG has not yet been found in the literature for vocal-music teaching and performance evaluation. In this study, 16 music students were invited to participate in a 2-stage experiment. The two-stage experiment was designed to collect data for the two teaching methods: self-imagination and VR training. The two methods were compared by analyzing the three types of data: self-rating reports, third-party evaluation, and EEG emotion classification data. An empirical judgment of the study is, as long as we elicit the emotional state corresponding to the song emotion (such as the positive song corresponds to the positive emotion), the singing effect will be improved; the better the effect of the emotional elicitation, the more obvious the improvement of the singing effect. Therefore, this study aimed to verify the hypothesis that VR can better elicit the positive, neutral, and negative emotions states than self-imagination, and further improve the singing performance due to the improvement of emotional activation.

## Methods

**FIGURE 1:**
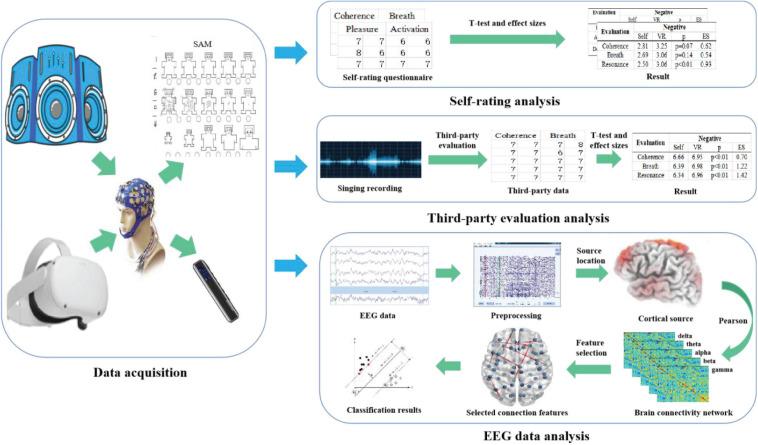
The flow chart of the empirical study.

Figure 1 shows a flowchart of the proposed method, mainly including data collection and data analysis. The data collection part collected the EEG data, self-rating reports, and third-party evaluation data. The data analysis part conducted a statistical investigation on self-rating reports, third-party evaluation data, and emotion recognition based on functional connectivity features of EEG data.

### Experiment and Data Collection

#### Participants

In this empirical study, 16 college students (8 male students and 8 female students, mean age of 19.5 ± 1.54) from the Art School of Nanjing University of Aeronautics and Astronautics were invited to participate in this experiment. All participants were right-handed and have normal hearing, normal vision, or vision correction, and no brain or mental illness. They were asked to maintain adequate sleep time before the experiment. They agreed and signed a written informed consent form. The experiment and data in this empirical study complied with all relevant ethical laws and regulations. Ethical guidelines were followed for conducting experiments with human participants.

#### The Description of the Designed Experiment

Six songs in three emotion categories (positive, neutral, and negative) were selected as emotional stimulation materials. Each emotion category has been assigned by two songs. Each song was edited into a 3-min core segment, the lyrics of each song were removed, and only the background music was retained. The emotional category tags and song names of the selected songs are shown in Table 1.

**TABLE 1:** Emotional stimulation materials.

Order	Emotion category	Translated name of song	Original name of song
1	Negative	Mama in the candlelight	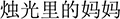
2	Negative	Where does the time go	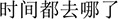
3	Neutral	Pastoral song	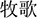
4	Neutral	By Lake Baikal	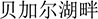
5	Positive	My motherland and me	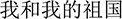
6	Positive	In the field of hope	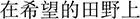

**FIGURE 2:**
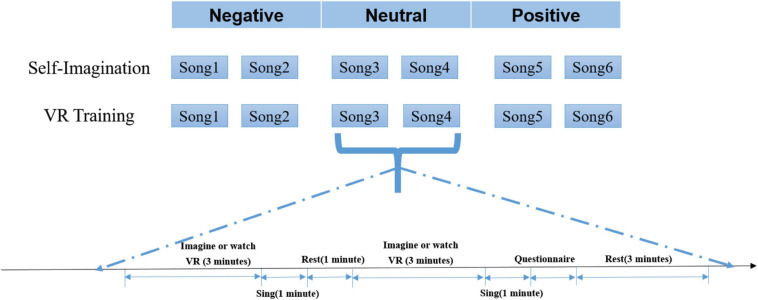
Experimental paradigm.

In the experiment, each subject was asked to participate in a two-stage experiment on the emotion elicited by the self-imagination and VR training. The complete experimental paradigm is shown in Figure 2. In the self-imagination stage, the vocal music teaching scene was simulated, the edited songs were played, and the subjects were allowed to imagine themselves according to the songs they heard, and mobilized the scenes, and elicited emotions required for singing. In the VR training stage, participants were asked to wear VR glasses (Quest2, Oculus, United States) and watched VR videos made according to the emotional background of the song. To better simulate the real vocal music teaching scene, in the self-imagining stage, a teaching guide commentary was added before the background music of the song was played, which introduced the relevant background of the song and the emotion required for the singing scene of the song. However, the background music of the VR training video was only the background music that the edited song played in the VR training.

For each emotion category, each stage of the experiment was divided into three groups, negative, neutral, and positive. And each group included an experiment of two songs in the same emotion category. The experimental process of each song included self-imagination and watching a VR video for 3 min to elicit emotion in singing. The subject sang for 1 min and the singing was recorded for evaluating the subject’s singing performance. Then the subject was asked to rest for 1 min and continued the experiment of the next song. After completing the experiment of two songs in each group, the subject was asked to fill out two self-rating reports: Self-Assessment Manikin (SAM) emotion self-rating form (Bradley and Lang, 1994), and vocal performance self-rating form. The two songs of each group were played in the order of negative, neutral, and positive, with a 3-min rest between each group. Participants rested for 10 min after the end of the three groups in each stage. In the experiment, the subjects were asked to stay as still as possible to prevent the influence of artifacts on the EEG. After all the experiments of a subject were completed, third-party evaluators evaluated the 12 songs according to the different dimensions of vocal singing (2 scenes × 6 songs) and gave a score based on the subject’s singing performance. The evaluators were five selected professional vocal teachers.

The SAM self-rating scale was set from one to nine points and used to measure emotional pleasure, activation, and dominance. The higher the score, the stronger the emotion indicating higher pleasure, higher arousal, and higher dominance. The vocal self-rating form provides self-rating scores on singing performance from seven aspects: coherence of song singing, use of breath, use of resonance, intonation, rhythm, language use, musicality, and emotional expression. A rubric with four levels for the assessment is used: excellent, good, medium, and poor. The corresponding numbers were 4, 3, 2, and 1. The higher the score, the better the singing performance. As for the third-party evaluation, the same seven aspects used for the vocal self-rating form were evaluated except the scale was set to from 1 to 10 points. The higher the score, the better the singing performance.

#### The Description of the EEG Recording

A 64-channel portable wireless EEG system (NeuSen.W64, Neuracle, China) was used for EEG data collection, and the sampling rate was set to 1,000 Hz. According to the international standard 10–20 system, the EEG data were recorded of 59 electrodes, AF3/4, AF7/8, Fp1/2, Fz, F1/2, F3/4, F5/6, F7/8, FC1/2, FC3/4, FC5/6, FCz, Cz, C1/2, C3/4, C5/6, FT7/8, T7/8, TP7/8, CP1/2, CP3/4, CP5/6, FPz, Pz, P3/4, P5/6, P7/8, PO3/4, PO5/6, PO7/8, POz, Oz, O1/2, with CPz as the reference electrode and AFz as the ground electrode. Throughout the experiment, the impedance of all electrodes was kept below 5 kΩ.

### Experimental Method

#### EEG Signal Preprocessing

In this study, the EEGLAB (Delorme and Makeig, 2004) toolbox was used to preprocess the EEG data. After channel location, the original EEG data was band-pass filtered at 1–45 Hz, the whole brain was averaged re-referenced, and down-sampled to 250 Hz. Then, independent component analysis was used to decompose the EEG signal into independent components, and the ICLabel (Pion-Tonachini et al., 2019) was used to remove artifacts such as eye movement and muscle movement. After preprocessing, the last 150 s EEG signal of each song was divided into 1 s period EEG signal as a sample to obtain the maximum emotional response (Li et al., 2020).

#### Source Location and Functional Connectivity Estimation

Due to the volume conduction effect, the brain activation area derived from the scalp EEG is not accurate, and the electrical signals from the brain can be recorded from multiple nearby sensors (Dimitrakopoulos et al., 2017). Therefore, a brain network calculation method was used similar to some literature described (Dimitrakopoulos et al., 2017; Wang et al., 2020). First, the source localization brain activation of each frequency band was calculated, and then the cortical functional network was calculated. The Brainstorm software (Tadel et al., 2011) for source location was used. The ICBM152 template was used to construct the head model and source space, and map the scalp electrodes to the ICBM152 (T1) head model. The head model used in this study was divided into three tissue types: scalp, brain, and skull. Their default conductivity was 1, 0.0125, and 1 s/m, respectively. The direction of each source was restricted to be perpendicular to the surface of the cortex, and the Boundary Element Method (BEM) (Mosher and Leahy, 1999) was used to calculate the guiding field matrix L. The cerebral cortex source signal was reconstructed by standardized low-resolution electromagnetic tomography (sLORETA) (Pascual-Marqui, 2002), the direction was fixed and perpendicular to the cortex, and all the parameters of sLORETA uses the default settings.

According to the Desikan-Killiany map (Desikan et al., 2006), the cerebral cortex gray matter was divided into 68 regions. The Pearson correlation coefficient was used to construct functional connectivity, which has been widely used in functional magnetic resonance imaging research (Dimitrakopoulos et al., 2017). The source signal was split into five frequency bands (delta: 1–4 HZ, theta: 4–8 HZ, alpha: 8–13 HZ, beta: 13–30 HZ, and gamma: 30–45 HZ), for the source of each frequency band in each area. The signals were averaged to obtain the source signal of the brain region. The functional network was calculated by the Pearson correlation coefficient between the source signals of each brain region pair. Each sample got 11,390 functional connectivity features for classification, that was, 68 × (68−1)/2 = 2,278 features for each frequency band.

#### Feature Selection and Emotion Classification

In this empirical study, emotions were divided into negative, neutral, and positive. For each sample, the source functional connectivity features were extracted separately, and different tags were marked according to different emotional types of songs.

The Max-relevance and min-redundancy (mRMR) algorithm (Peng et al., 2005) was used for feature selection and select the most important 10 features for emotion classification. The algorithm uses mutual information to measure the relationship between features and categories. It maximized the correlation between features and categorical variables and minimized the redundancy between features. In other words, the algorithm found m features from the feature space that has the greatest correlation with the target category and the least redundancy with other features. The definition of maximum correlation is shown in Eq. 1, and the definition of minimum redundancy is shown in Eq. 2:

(1) $$m\,a\,x\,D\,\left(S,c\right)=\frac{1}{\left|S\right|}\,\sum_{f_{i}\in S}I\,\left(f_{i};c\right)$$

(2) $$m\,i\,n\,R\,\left(S\right)=\frac{1}{{\left|S\right|}^{2}}\,\sum_{f_{i},f_{j}\in S}I\,\left(f_{i};f_{j}\right)$$

where S represents the feature set; c represents the target category; *I*(*f*_*i*_; *c*)represents the mutual information between the feature i and the target category c; *I*(*f*_*i*_; *f*_*j*_) is the mutual information between feature i and feature j.

The support vector machine (SVM) with a radial basis function (RBF) kernel was used for emotion recognition. As a widely used classifier, SVM has been proved by a large number of studies to be a practical EEG classification method (Bashivan et al., 2015). The 10-fold cross-validation was used to calculate the accuracy of the classification. The SVM kernel function parameters g (0.5–4, step size 0.5) and penalty coefficient c (10^–2^ to 10^2^, step size coefficient is 10) were traversed to get the optimal parameters. To explore the influence of functional connectivity features of different frequency bands on the results, the functional connectivity features of 5 frequency bands in the 2 scenarios were, respectively, cross-verified 100 times. The five frequency band features were classified separately and the five frequency band features were spliced into a feature vector for classification.

#### Statistical Method

A statistical analysis of three rating scales was performed, including SAM self-rating report, vocal performance self-rating report, and third-party evaluation scores. Given different emotion categories, the three rating scales of emotion data in both self-imagination and VR training methods were gone through the test by *t*-test and effect sizes (Lakens, 2013). The detailed results are described in the following section.

## Results

### SAM Self-Rating

The SAM self-rating data were collected from the 16 subjects, from the perspective of emotional pleasure, activation and dominance. The average score and statistical test results of SAM are shown in Table 2. In the case of negative emotion, participants reported lower evaluations of VR training (*M* = 3.00) than self-imagination (*M* = 4.69), *p* < 0.01, 95%CI [1.02, 2.35], Hedges’s *g* = 1.27 for pleasure; higher evaluations of VR training (*M* = 7.00) than self-imagination (*M* = 5.56), *p* < 0.01, 95%CI [0.85, 2.02], Hedges’s *g* = 1.03 for activation; lower evaluations of VR training (*M* = 4.63) than self-imagination (*M* = 6.06), *p* < 0.01, 95%CI [0.61, 2.26], Hedges’s *g* = 0.89 for dominance. Under neutral emotion, participants reported higher evaluations of VR training (*M* = 6.19) than self-imagination (*M* = 5.94), *p* = 0.43, 95%CI [−0.41, 0.91], Hedges’s *g* = 0.18 for pleasure; higher evaluations of VR training (*M* = 6.13) than self-imagination (*M* = 5.69), *p* = 0.34, 95%CI [−0.52, 1.39], Hedges’s *g* = 0.29 for activation; higher evaluations of VR training (*M* = 6.69) than self-imagination (*M* = 6.50), *p* = 0.59, 95%CI [−0.55, 0.92], Hedges’s *g* = 0.14 for dominance. Under positive emotion, participants reported higher evaluations of VR training (*M* = 8.06) than self-imagination (*M* = 7.00), *p* < 0.01, 95%CI [0.57, 1.56], Hedges’s *g* = 1.13 for pleasure; higher evaluations of VR training (*M* = 7.56) than self-imagination (*M* = 6.31), *p* < 0.01, 95%CI [0.89, 1.61], Hedges’s *g* = 1.42 for activation; lower evaluations of VR training (*M* = 6.06) than self-imagination (*M* = 6.44), *p* = 0.33, 95%CI [−0.42, 1.17], Hedges’s *g* = 0.22 for dominance.

**TABLE 2:** Average score and statistical test results of Self-Assessment Manikin (SAM).

Evaluation	Negative				Neutral				Positive			
	Self	VR	*p*	ES	Self	VR	*p*	ES	Self	VR	*p*	ES
Pleasure	4.69	3.00	*p* < 0.01	1.27	5.94	6.19	*p* = 0.43	0.18	7.00	8.06	*p* < 0.01	1.13
Activation	5.56	7.00	*p* < 0.01	1.03	5.69	6.13	*p* = 0.34	0.29	6.31	7.56	*p* < 0.01	1.42
Dominance	6.06	4.63	*p* < 0.01	0.89	6.50	6.69	*p* = 0.59	0.14	6.44	6.06	*p* = 0.33	0.22

### Self-Rating on Vocal Performance

The vocal self-rating data were collected from the 16 subjects, and the score range was set to 1–4. The average scores and statistical test results of the scale are shown in Table 3. In the self-rating scale of negative emotion, participants reported higher evaluations of VR training (*M* = 3.25) than self-imagination (*M* = 2.81), *p* = 0.07, 95%CI [−0.04, 0.91], Hedges’s *g* = 0.62 for coherence; higher evaluations of VR training (*M* = 3.06) than self-imagination (*M* = 2.69), *p* = 0.14, 95%CI [−0.14, 0.89], Hedges’s *g* = 0.54 for breath; higher evaluations of VR training (*M* = 3.06) than self-imagination (*M* = 2.50), *p* < 0.01, 95%CI [0.23, 0.90], Hedges’s *g* = 0.93 for resonance; higher evaluations of VR training (*M* = 3.06) than self-imagination (*M* = 2.94), *p* = 0.54, 95%CI [−0.30, 0.55], Hedges’s *g* = 0.20 for intonation; higher evaluations of VR training (*M* = 3.06) than self-imagination (*M* = 2.94), *p* = 0.43, 95%CI [−0.20, 0.45], Hedges’s *g* = 0.25 for language; higher evaluations of VR training (*M* = 3.44) than self-imagination (*M* = 3.16), *p* = 0.06, 95%CI [−0.01, 0.63], Hedges’s *g* = 0.50 for musicality; higher evaluations of VR training (*M* = 3.31) than self-imagination (*M* = 2.75), *p* < 0.01, 95%CI [0.23, 0.90], Hedges’s *g* = 0.88 for expression. In the self-rating scale for neutral emotion, participants reported higher evaluations of VR training (*M* = 3.38) than self-imagination (*M* = 3.25), *p* = 0.43, 95%CI [−0.20, 0.45], Hedges’s *g* = 0.16 for coherence; higher evaluations of VR training (*M* = 3.38) than self-imagination (*M* = 3.19), *p* = 0.19, 95%CI [−0.10, 0.48], Hedges’s *g* = 0.29 for breath; higher evaluations of VR training (*M* = 3.25) than self-imagination (*M* = 2.88), *p* = 0.05, 95%CI [−0.01, 0.76], Hedges’s *g* = 0.63 for resonance; higher evaluations of VR training (*M* = 3.31) than self-imagination (*M* = 3.13), *p* = 0.27, 95%CI [−0.16, 0.54], Hedges’s *g* = 0.26 for intonation; higher evaluations of VR training (*M* = 3.38) than self-imagination (*M* = 3.06), *p* = 0.06, 95%CI [−0.01, 0.63], Hedges’s *g* = 0.59 for language; higher evaluations of VR training (*M* = 3.56) than self-imagination (*M* = 3.44), *p* = 0.33, 95%CI [−0.14, 0.39], Hedges’s *g* = 0.22 for musicality; higher evaluations of VR training (*M* = 3.44) than self-imagination (*M* = 3.00), *p* < 0.01, 95%CI [0.10, 0.77], Hedges’s *g* = 0.76 for expression. In the self-rating scale of positive emotion, participants reported higher evaluations of VR training (*M* = 3.44) than self-imagination (*M* = 3.31), *p* = 0.34, 95%CI [−0.14, 0.39], Hedges’s *g* = 0.23 for coherence; higher evaluations of VR training (*M* = 3.31) than self-imagination (*M* = 3.13), *p* = 0.19, 95%CI [−0.10, 0.48], Hedges’s *g* = 0.31 for breath; higher evaluations of VR training (*M* = 3.06) than self-imagination (*M* = 3.19), *p* = 0.5, 95%CI [−0.26, 0.51], Hedges’s *g* = 0.22 for resonance; higher evaluations of VR training (*M* = 3.00) than self-imagination (*M* = 2.69), *p* = 0.14, 95%CI [−0.11, 0.74], Hedges’s *g* = 0.44 for intonation; higher evaluations of VR training (*M* = 3.31) than self-imagination (*M* = 3.00), *p* < 0.05, 95%CI [0.06, 0.57], Hedges’s *g* = 0.92 for language; lower evaluations of VR training (*M* = 3.31) than self-imagination (*M* = 3.38), *p* = 0.75, 95%CI [−0.35, 0.47], Hedges’s *g* = 0.09 for musicality; higher evaluations of VR training (*M* = 3.63) than self-imagination (*M* = 3.25), *p* < 0.05, 95%CI [0.05, 0.70], Hedges’s *g* = 0.63 for expression.

**TABLE 3:** Average scores and statistical test results of the self-rating.

Evaluation	Negative				Neutral				Positive			
	Self	VR	*p*	ES	Self	VR	*p*	ES	Self	VR	*p*	ES
Coherence	2.81	3.25	*p* = 0.07	0.62	3.25	3.38	*p* = 0.43	0.16	3.31	3.44	*p* = 0.34	0.23
Breath	2.69	3.06	*p* = 0.14	0.54	3.19	3.38	*p* = 0.19	0.29	3.13	3.31	*p* = 0.19	0.31
Resonance	2.50	3.06	*p* < 0.01	0.93	2.88	3.25	*P* = 0.05	0.63	3.06	3.19	*p* = 0.50	0.22
Intonation	2.94	3.06	*p* = 0.54	0.20	3.13	3.31	*p* = 0.27	0.26	2.69	3.00	*p* = 0.14	0.44
Language	2.94	3.06	*p* = 0.43	0.25	3.06	3.38	*p* = 0.06	0.59	3.00	3.31	*p* < 0.05	0.92
Musicality	3.16	3.44	*p* = 0.06	0.50	3.44	3.56	*p* = 0.33	0.22	3.38	3.31	*p* = 0.75	0.09
Expression	2.75	3.31	*p* < 0.01	0.88	3.00	3.44	*p* < 0.01	0.76	3.25	3.63	*p* < 0.05	0.63

*We rename the performance from seven aspects as coherence, breath, resonance, intonation, language, musicality, and expression, respectively.*

### Third-Party Evaluation

The third-party evaluation data on singing performance were collected from the five professional vocal teachers. The average score of the subjects and the results of statistical test are shown in Table 4. Under negative emotion, participants reported higher evaluations of VR training (*M* = 6.95) than self-imagination (*M* = 6.66), *p* < 0.01, 95%CI [0.07, 0.50], Hedges’s *g* = 0.70 for coherence; higher evaluations of VR training (*M* = 6.98) than self-imagination (*M* = 6.39), *p* < 0.01, 95%CI [0.38, 0.80], Hedges’s *g* = 1.22 for breath; higher evaluations of VR training (*M* = 6.96) than self-imagination (*M* = 6.34), *p* < 0.01, 95%CI [0.33, 0.92], Hedges’s *g* = 1.42 for resonance; higher evaluations of VR training (*M* = 6.26) than self-imagination (*M* = 6.18), *p* = 0.18, 95%CI [−0.11, 0.29], Hedges’s *g* = 0.16 for intonation; lower evaluations of VR training (*M* = 6.58) than self-imagination (*M* = 6.66), *p* = 0.07, 95%CI [−0.04, 0.21], Hedges’s *g* = 0.17 for language; higher evaluations of VR training (*M* = 7.34) than self-imagination (*M* = 6.58), *p* < 0.01, 95%CI [0.50, 1.02], Hedges’s *g* = 1.52 for musicality; higher evaluations of VR training (*M* = 7.81) than self-imagination (*M* = 6.61), *p* < 0.01, 95%CI [0.95, 1.45], Hedges’s *g* = 2.21 for expression. Under neutral emotion, participants reported higher evaluations of VR training (*M* = 7.00) than self-imagination (*M* = 6.73), *p* < 0.01, 95%CI [0.08, 0.47], Hedges’s *g* = 0.83 for coherence; higher evaluations of VR training (*M* = 7.13) than self-imagination (*M* = 6.65), *p* < 0.01, 95%CI [0.23, 0.72], Hedges’s *g* = 1.09 for breath; higher evaluations of VR training (*M* = 7.38) than self-imagination (*M* = 6.58), *p* < 0.01, 95%CI [0.46, 1.14], Hedges’s *g* = 1.42 for resonance; higher evaluations of VR training (*M* = 6.36) than self-imagination (*M* = 6.29), *p* = 0.22, 95%CI [−0.13, 0.28], Hedges’s *g* = 0.14 for intonation; lower evaluations of VR training (*M* = 6.63) than self-imagination (*M* = 6.64), *p* = 0.43, 95%CI [−0.15, 0.17], Hedges’s *g* = 0.03 for language; higher evaluations of VR training (*M* = 7.35) than self-imagination (*M* = 6.89), *p* < 0.01, 95%CI [0.19, 0.73], Hedges’s *g* = 1.02 for musicality; higher evaluations of VR training (*M* = 7.73) than self-imagination (*M* = 6.98), *p* < 0.01, 95%CI [0.54, 0.96], Hedges’s *g* = 1.49 for expression. Under positive emotion, participants reported higher evaluations of VR training (*M* = 7.10) than self-imagination (*M* = 6.80), *p* < 0.01, 95%CI [0.12, 0.48], Hedges’s *g* = 0.74 for coherence; higher evaluations of VR training (*M* = 7.08) than self-imagination (*M* = 6.60), *p* < 0.01, 95%CI [0.27, 0.68], Hedges’s *g* = 0.90 for breath; higher evaluations of VR training (*M* = 7.39) than self-imagination (*M* = 6.56), *p* < 0.01, 95%CI [0.58, 1.07], Hedges’s *g* = 1.32 for resonance; higher evaluations of VR training (*M* = 6.45) than self-imagination (*M* = 6.38), *p* = 0.20, 95%CI [−0.11, 0.26], Hedges’s *g* = 0.13 for intonation; lower evaluations of VR training (*M* = 6.68) than self-imagination (*M* = 6.69), *p* = 0.42, 95%CI [−0.12, 0.14], Hedges’s *g* = 0.03 for language; higher evaluations of VR training (*M* = 7.41) than self-imagination (*M* = 6.70), *p* < 0.01, 95%CI [0.51, 0.91], Hedges’s *g* = 1.38 for musicality; higher evaluations of VR training (*M* = 7.75) than self-imagination (*M* = 6.71), *p* < 0.01, 95%CI [0.78, 1.30], Hedges’s *g* = 2.02 for expression.

**TABLE 4:** Average score of the subjects and the statistical test results of the third-party evaluation.

Evaluation	Negative				Neutral				Positive			
	Self	VR	*p*	ES	Self	VR	*p*	ES	Self	VR	*p*	ES
			
Coherence	6.66	6.95	*p* < 0.01	0.70	6.73	7.00	*p* < 0.01	0.83	6.80	7.10	*p* < 0.01	0.74
Breath	6.39	6.98	*p* < 0.01	1.22	6.65	7.13	*p* < 0.01	1.09	6.60	7.08	*p* < 0.01	0.90
Resonance	6.34	6.96	*p* < 0.01	1.42	6.58	7.38	*p* < 0.01	1.42	6.56	7.39	*p* < 0.01	1.32
Intonation	6.18	6.26	*p* = 0.18	0.16	6.29	6.36	*p* = 0.22	0.14	6.38	6.45	*p* = 0.20	0.13
Language	6.66	6.58	*p* = 0.07	0.17	6.64	6.63	*p* = 0.43	0.03	6.69	6.68	*p* = 0.42	0.03
Musicality	6.58	7.34	*p* < 0.01	1.52	6.89	7.35	*p* < 0.01	1.02	6.70	7.41	*p* < 0.01	1.38
Expression	6.61	7.81	*p* < 0.01	2.21	6.98	7.73	*p* < 0.01	1.49	6.71	7.75	*p* < 0.01	2.02

### Emotion Classification

In the 2 scenarios of self-imagination and VR training, based on the top 10 features selected by the mRMR algorithm, Table 5 shows the emotion classification accuracy using the SVM under the optimal parameters (the average of 1,000 classification accuracy). All bands mean directly splicing all the features of five frequency bands together. In the results of six cases, the emotion classification accuracy of VR training was greater than the accuracy of self-imagining. In the self-imagination scenario, the highest classification accuracy was 82.82% based on all frequency bands. In a VR training scenario, the highest emotion classification accuracy was 85.84% acquired with the gamma frequency band. The best results were bold in Table 5.

**TABLE 5:** Classification accuracy of emotion recognition.

Cases	Delta	Theta	Alpha	Beta	Gamma	All bands
Self-imagination	43.00	47.75	53.00	79.05	80.47	**82.82**
VR training	45.68	51.80	55.55	80.55	**85.84**	84.69

### Selected Connectivity Features

**FIGURE 3:**
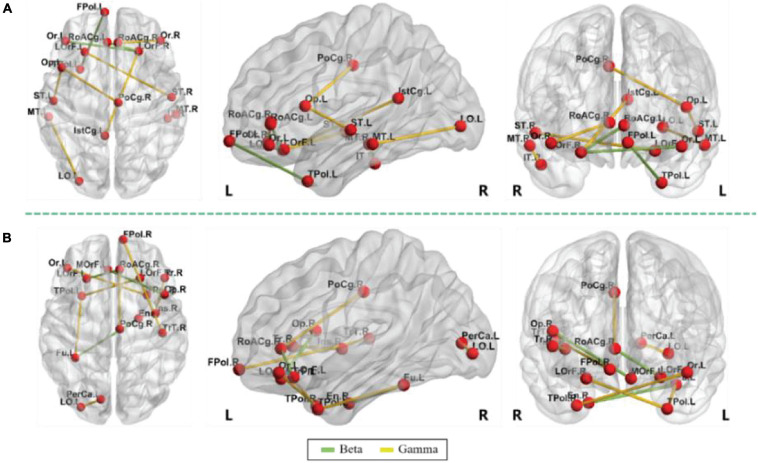
Discriminative top 10 features, panel **(A)** is self-imagination and panel **(B)** is virtual reality (VR) training.

This section showed the top 10 most important functional connectivity features selected by the mRMR algorithm in the 2 scenarios and displays them in the form of charts. The specific functional connectivity features and corresponding brain areas are shown in Figure 3 and Table 6. The abbreviations of these brain areas were adopted from Román et al. (2017) study. It can be seen from Table 6 that the functional connectivity features were only distributed in the beta and gamma frequency bands. In both scenarios, three functional connectivity features were located in the beta frequency band and the rest seven were in the gamma frequency band. Also, the asymmetry between the cerebral hemispheres was observed. In the self-imagination scenario, there were 10 brain areas in the right hemisphere and 7 brain areas in the left hemisphere; in the VR training scenario, there were 7 brain areas in the right hemisphere and 10 brain areas in the left hemisphere. Besides, the connectivity features between the two hemispheres were strong. In both scenarios, there were five connectivity features between the hemispheres.

**TABLE 6:** Discriminative top 10 functional connectivity features.

Cases	Selected features
Self-imagination	**Beta:** frontal pole L–temporal pole L (FPol L–TPol L), lateral orbitofrontal R–pars orbitalis L (LOrF R–Or L), lateral orbitofrontal R–rostral anterior cingulate L (LOrF R–RoACg L).
	**Gamma:** inferior temporal R–middle temporal R (IT R–MT R), isthmus cingulate L–lateral orbitofrontal R (IstCg L–LOrF R), lateral occipital L–middle temporal L (LO L–MT L), lateral orbitofrontal L–superior temporal R (LOrF L–ST R), pars opercularis L–posterior cingulate R (Op L–PoCg R), pars opercularis L–superior temporal L (Op L–ST L), pars orbitalis R–rostral anterior cingulate R (Or R–RoACg R).
VR training	**Beta:** entorhinal R–fusiform L (En R–Fu L), lateral orbitofrontal L–rostral anterior cingulate R (LOrF L–RoACg R), medial orbitofrontal L–pars opercularis R (MOrF L–Op R).
	**Gamma:** frontal pole R–transverse temporal R (FPol R–TrT R), fusiform L–temporal pole L (Fu L–TPol L), insula R–pars triangularis R (Ins R–Tr R), lateral occipital L–pericalcarine L (LO L–PerCa L), lateral orbitofrontal R–temporal pole L (LOrF R–TPol L), pars orbitalis L–temporal pole R (Or L–TPol R), posterior cingulate R–rostral anterior cingulate R (PoCg R–RoACg R).

## Discussion

This empirical study conducted a statistical test on self-rating report data and third-party teacher evaluation data for the two methods: self-imagination and VR training. Emotion classification based on the EEG functional connectivity features was also conducted. From the perspective of self-rating reports and third-party evaluation, the differences in an emotional state and singing performance and the differences in functional connectivity were compared and revealed. There were significant differences between the two methods to some extent. The VR training got higher evaluation scores than self-imagination. The emotion classification accuracy in the VR training was higher than that of the self-imagination. These results supported this empirical study’s initial hypothesis that compared with the traditional self-imagination method, VR training can better elicit the emotional state of the singers, further improve their singing performance, and provide a new teaching aid method for vocal music teaching.

### Self-Rating Scale

In this empirical study, the SAM scale and the vocal self-rating scale were used to rate the emotional state and singing performance. As shown in Table 2, regarding the self-rating scales under the negative emotion and the positive emotion, there were significant differences between the self-imagination and the VR training in terms of pleasure and activation. The average scores were in line with the expectations, that is, the VR training under the negative emotion reduced pleasure, while under the positive emotion, it increased pleasure. And, the VR training under both negative and positive increased activation. However, in terms of emotional dominance, there were significant differences between the two methods under the negative emotion, and the VR training reduced dominance, while under the positive emotion, there was no significant difference between the two methods (*p* = 0.33), this may be because VR training under the negative emotion was easier to achieve good emotion-elicited effects (Liao et al., 2019). Since the valence and arousal in the neutral emotion were generally medium, there was no significant difference in terms of pleasure, arousal, and dominance for the two methods.

It can be seen from Table 3, regarding the self-rating scale for a vocal performance, there were significant differences in the scores of emotional expression between the two methods. And the average score of the VR training was higher than the average score of self-imagination, indicating that the improvement of emotional activation by VR training could be applied well in the singers’ singing emotion.

Regarding the evaluation of third-party professional vocal teachers on singing performance, Table 4 showed that in terms of the singing coherence, breath use, resonance use, musical sense, and emotional expression, the singing performance score after VR training was significantly higher than that of the self-imagination. This could be due to that the VR training transmitted emotional information to the subjects in an audio-visual way and enhanced the subjects’ emotional state. The emotional information in a relaxed and natural state improved the subjects’ singing level. In terms of pronouncing words and intonation rhythm, there was no significant difference between the two methods. The correct pronouncing of words is the premise of language purity and could have an impact on the performance of vocal art (Li, 2016). Singing pronouncing words vary from person to person. The participants in the experiment were all students who have received systematic vocal training. They have undergone long-term professional training in pronouncing words and intonation rhythm. The singing songs had been practiced before the subjects participated in the experiment, and the correct intonation rhythm had also been mastered. It would not be significantly changed in a short time through third-party evaluation data on singing performance.

### Emotion-Related Functional Connectivity

This empirical study classified emotion from the two methods based on the functional connectivity features after source location. The accuracy of emotion classification under the VR training was greater than the self-imagination. Among the five frequency bands, the beta and gamma frequency bands had high classification accuracy, and the top 10 most important functional connectivity features selected were all located in the beta and gamma frequency bands. This was consistent with previous studies, indicating that the beta and gamma bands of brain activity are more related to emotional processing than other frequency bands (Zheng and Lu, 2015; Zheng et al., 2017; Li et al., 2018).

Among the functional connectivity features for the self-imagination method, the right lateral orbitofrontal was related to both beta and gamma bands, which was consistent with the findings of previous studies. The lateral prefrontal cortex controls the experience and expression of emotion, especially playing an important role in the processing of negative emotion (Hooker and Knight, 2006). There were also four brain regions in the beta frequency band, including the left frontal pole, left temporal pole, left pars orbitalis, and left rostral anterior cingulate. There were 13 brain regions in the gamma band, right inferior temporal, left and right middle temporal, left isthmus cingulate, left lateral occipital, left lateral orbitofrontal, left and right superior temporal, left pars opercularis, right posterior cingulate, right pars orbitalis, right rostral anterior cingulate. The prefrontal cortex played an important role in emotional processing (Esslen et al., 2004). The temporal cortex was involved in the processing of humor, laughter, and smiles (Wild et al., 2006). The rostral anterior cingulate had a regulatory effect on the brain regions that produce emotional responses and can be enhanced by emotional valence (Chiu et al., 2008). The pars opercularis played a role in regulating the perception of emotional rhythm (Patel et al., 2018). Emotional stimulation activated the posterior cingulate cortex (Maddock et al., 2003) and the pars orbitalis (Krautheim et al., 2020).

Among the functional connectivity features for the VR training method, the left fusiform and the right rostral anterior cingulate were related to both beta and gamma bands. This could be due to the fusiform provided perception for participants in VR scenes to detect the difference between the virtual environment and the real physical world (Garcia et al., 2012), and the right rostral anterior cingulate regulated emotion (Chiu et al., 2008; Alvarez et al., 2011). There were also five brain regions in the beta band, including the right entorhinal, left lateral orbitofrontal, right rostral anterior cingulate, and left medial orbitofrontal, and right pars opercularis. There were 13 brain regions in the gamma band, including the left frontal pole, right transverse temporal, left temporal pole, right insula, and right inferior frontal pole, right pars triangularis, left lateral occipital, left pericalcarine, right lateral orbitofrontal, left pars orbitalis, right temporal pole, and right posterior cingulate. The entorhinal cortex was a key neural structure for spatial navigation (Jacobs et al., 2010). Many studies have studied the activation and changes of the entorhinal cortex in the VR environment (Jacobs et al., 2010; Howett et al., 2019). The medial frontal cortex (including the medial orbitofrontal cortex) played a crucial role in the processing of negative emotion (Sitaram et al., 2011). Insula activation involves emotional processing, and studies have shown that the posterior and anterior sub-regions of the human insula showed obvious response characteristics to auditory emotional stimuli (Zhang Y. et al., 2019).

Seven brain regions existed in the features selected for the two methods, including the right rostral anterior cingulate, left lateral occipital, right lateral orbitofrontal, left pars orbitalis, right posterior cingulate, left lateral orbitofrontal, and left temporal pole, were all related to the activation and processing of emotion (Maddock et al., 2003; Esslen et al., 2004; Wild et al., 2006; Güntekin and Basar, 2007; Chiu et al., 2008; Krautheim et al., 2020). The brain regions and functional connectivity features involved in this research provided references for research on brain emotion activation mechanisms, the impact of VR on the brain, and EEG-based emotion recognition.

### Limitations

One limitation of the empirical study is that the common template (ICBM152) was not used to obtain the head model and source space representation of all participants. Studies have shown that using an individualized template to construct a head model and source space for each participant can obtain more accurate results in the source location process (Lei et al., 2011; Hassan and Wendling, 2018). The individualized template helps further improve the performance of emotion recognition and discover more physiologically relevant features. Other network analysis methods, such as Phase Lock Value (PLV) (Li et al., 2019), Phase Slope Index (PSI) (Basti et al., 2018), Partially Directed Coherence (PDC) (Wang et al., 2020) can also be used for functional network analysis after source location. Therefore, individualized templates will be used in future work to establish a head model in source space, and other functional connectivity construction methods will be used to improve EEG-based emotion recognition.

This study demonstrated the utility of VR for eliciting emotions to trainees-singers and further improving their performance. However, the cost of VR equipment may still be unaffordable for some institutions with a limited budget. Another limitation is that EEG signals may be easily disturbed by singing and/or moving actions. Finally, the sample size of this empirical study was relatively small. Future attempts should strive to address these limitations and collect data from a larger sample.

Furthermore, the adverse symptoms and effects (i.e., cybersickness) during VR training may undermine the health and safety standards, and compromise the reliability of the experimental results (Kourtesis et al., 2019a). The current study did not examine the symptoms of cybersickness, while they may negatively affect cognitive and behavioral performance, as well as EEG data (Kourtesis et al., 2019a; Weech et al., 2019). Also, this empirical study did not examine the immersive user experience. These factors play a central role in the efficiency of the VR experience (Slater, 2009; Skarbez et al., 2017) and the incidence of cybersickness (Kourtesis et al., 2019b; Weech et al., 2019). Future attempts should consider the administration of recently published cybersickness questionnaires (Somrak et al., 2021).

## Conclusion

In this study, a VR training task was implemented to elicit emotions from singers and assist them with further improving their emotional singing performance. The VR training method was compared against the traditional self-imagination method. By conducting a two-stage experiment, the two methods were compared in terms of emotions’ elicitation and emotional singing performance. In the first stage, electroencephalographic (EEG) data were collected from the subjects. In the second stage, self-rating reports and third-party teachers’ evaluations were collected. The EEG data were analyzed by adopting the max-relevance and min-redundancy algorithm for feature selection and the SVM for emotion recognition. The experimental results have validated that VR can better elicit the positive, neutral, and negative emotional states of singers than self-imagination, and further improve the singing performance due to the improvement of emotional activation. As such, we argue the VR training method can be seen as an effective approach that will improve and complement the available vocal music teaching methods.

## Data Availability Statement

The original contributions presented in the study are included in the article/supplementary material, further inquiries can be directed to the corresponding author/s.

## Ethics Statement

The studies involving human participants were reviewed and approved by the Ethics Committee of the Affiliated Nanjing Brain Hospital, Nanjing Medical University Nanjing. The patients/participants provided their written informed consent to participate in this study.

## Author Contributions

JZ and DZ proposed the research topic and outline. ZX, YZ, PW, and XX designed the procedures of experiments, collected, and analyzed the data. ZX, YZ, and PF contributed to the structuring and writing of the manuscript. All authors contributed to the article and approved the submitted version.

## Conflict of Interest

The authors declare that the research was conducted in the absence of any commercial or financial relationships that could be construed as a potential conflict of interest.

## Publisher’s Note

All claims expressed in this article are solely those of the authors and do not necessarily represent those of their affiliated organizations, or those of the publisher, the editors and the reviewers. Any product that may be evaluated in this article, or claim that may be made by its manufacturer, is not guaranteed or endorsed by the publisher.
